# Specimen-level phylogenetics in paleontology using the Fossilized Birth-Death model with sampled ancestors

**DOI:** 10.7717/peerj.3055

**Published:** 2017-03-01

**Authors:** Andrea Cau

**Affiliations:** Department of Earth, Life and Environmental Sciences, Alma Mater Studiorum, Bologna University, Bologna, Italy; Geological and Paleontological Museum “G. Capellini”, Bologna, Italy

**Keywords:** Bayesian phylogenetics, Morphological characters, Stratigraphy, Dipnoi, Lower Cretaceous, Specimen-level analysis, Tunisia, Fossilized Birth-Death model

## Abstract

Bayesian phylogenetic methods integrating simultaneously morphological and stratigraphic information have been applied increasingly among paleontologists. Most of these studies have used Bayesian methods as an alternative to the widely-used parsimony analysis, to infer macroevolutionary patterns and relationships among species-level or higher taxa. Among recently introduced Bayesian methodologies, the Fossilized Birth-Death (FBD) model allows incorporation of hypotheses on ancestor-descendant relationships in phylogenetic analyses including fossil taxa. Here, the FBD model is used to infer the relationships among an ingroup formed exclusively by fossil individuals, i.e., dipnoan tooth plates from four localities in the Ain el Guettar Formation of Tunisia. Previous analyses of this sample compared the results of phylogenetic analysis using parsimony with stratigraphic methods, inferred a high diversity (five or more genera) in the Ain el Guettar Formation, and interpreted it as an artifact inflated by depositional factors. In the analysis performed here, the uncertainty on the chronostratigraphic relationships among the specimens was included among the prior settings. The results of the analysis confirm the referral of most of the specimens to the taxa *Asiatoceratodus*, *Equinoxiodus, Lavocatodus* and *Neoceratodus*, but reject those to *Ceratodus* and *Ferganoceratodus*. The resulting phylogeny constrained the evolution of the Tunisian sample exclusively in the Early Cretaceous, contrasting with the previous scenario inferred by the stratigraphically-calibrated topology resulting from parsimony analysis. The phylogenetic framework also suggests that (1) the sampled localities are laterally equivalent, (2) but three localities are restricted to the youngest part of the section; both results are in agreement with previous stratigraphic analyses of these localities. The FBD model of specimen-level units provides a novel tool for phylogenetic inference among fossils but also for independent tests of stratigraphic scenarios.

## Introduction

The use of Bayesian inference methods in phylogenetic analysis of morphological features (Lewis, 2001; Nylander et al., 2004, see Lee & Palci, 2015) is a relatively novel approach in paleontology (Pyron, 2011; Lee et al., 2014a; Wright & Hillis, 2014; O’Reilly et al., 2016). In particular, co-estimation of topology and divergence times using morphology, including tip-dating methods (Ronquist et al., 2012), has become more popular in recent years, and it may represent a promising area for the integration of the two main sides of paleontology: the biostratigraphic (focusing on the distribution of the fossil record along the Geological Time) and the systematic (focusing on the inclusion of the fossil record in the Tree of Life). Stadler (2010) and Heath, Huelsenbeck & Stadler (2014) introduced a method for fossil calibration in phylogenetic analysis that integrates extinct and extant species with a single macroevolutionary model, named the “Fossilized Birth-Death (FBD) process” (Heath, Huelsenbeck & Stadler, 2014). Another significant area of application for Bayesian phylogenetic analyses is the reconstruction of evolutionary patterns among a set of taxa where both sister-taxon (cladogenetic) and ancestor-descendant (anagenetic) relationships are involved. In most of the studies mentioned above, the tree search strategies used were based on a strictly cladogenetic approach, which assumes that the analyzed ingroup does not include potential ancestors of other members of the same ingroup. Gavryushkina et al. (2014) introduced a Bayesian phylogenetic model that allows one sampled member of the analyzed ingroup to be a direct ancestor of another sampled taxon. This method, initially developed for analysis of molecular data, was implemented by Gavryushkina et al. (2016) allowing the inclusion of morphological data. As outlined by Gavryushkina et al. (2014) and Gavryushkina et al. (2016), failing to account for sampled ancestors may lead to significant bias in parameter estimation, in particular in nodal age inference, in the quantification of cladogenetic events and in the estimation of the fossil diversity.

The majority of the paleontological studies applying Bayesian phylogenetic methods and integrating the morphological and stratigraphic information of the terminal units included have focused on analysis of species-level taxa in order to reconstruct macroevolutionary patterns (e.g., Lee et al., 2014b; Close et al., 2015; Dembo et al., 2015; Fanti et al., 2015; Cau & Fanti, 2016; Bell et al., 2016; Fanti et al., 2016b). Specimen-level analysis (i.e., analysis using exclusively individual specimens as terminal tips) has been a poorly explored area of application of these new methodologies, compared to recent results that used parsimony as tree search strategy (e.g., Upchurch, Tomida & Barrett, 2004; Scannella et al., 2014; Mounier & Caparros, 2015; Tschopp, Mateus & Benson, 2015). Here, the FBD model implemented by Gavryushkina et al. (2016) is applied to the study on the affinities among specimen-level taxonomic operational units, specifically, dipnoan sarcopterygian specimens from the Lower Cretaceous Ain el Guettar Formation of southern Tunisia (Fanti et al., 2016a; Müller, 1844; Berg, 1940; Vorobiyeva, 1967). Recently, these specimens were analyzed integrating “traditional” stratigraphic, paleoecological and taphonomic methods with phylogenetic analysis of morphological features that used parsimony as tree search strategy (Fanti et al., 2016a). In that study, Fanti et al. (2016a) documented an unusually high diversity among the sample of isolated tooth plates, referable to five or more lineages (at genus-level, using Linnean-rank taxonomy) of dipnoans. The authors concluded that the high diversity of dipnoans in the Ain el Guettar Formation was a taphonomic artifact. In particular, Fanti et al. (2016a) suggested that a series of depositional factors significantly inflated observed lungfish diversity in the estuarine and marginal-marine deposits of the Oum ed Diab Member of the Ain el Guettar Formation, and concluded that the sampled fauna was representative of a larger, inland paleo-hydrographic system. Here, the data of Fanti et al. (2016a) is re-analyzed using Bayesian tip-dating approach for a discussion on the distribution of the dipnoan taxa across the four Tunisian localities sampled.

The aims of this study are to test (1) the application of the FBD model with sampled ancestors to a set of exclusively fossil taxa, (2) the use of Bayesian phylogenetic methods in specimen-level phylogenetics, (3) the incorporation of age uncertainty in phylogenetic models integrating both anagenetic and cladogenetic patterns, and (4) the application of phylogenetic models using both morphological and chronologic data as auxiliary tool for stratigraphic inference.

## Material and Methods

A discussion on the taxonomy and phylogenetic nomenclature of Mesozoic dipnoans (Müller, 1844; Berg, 1940; Vorobiyeva, 1967) is beyond the aims of this study. Furthermore, it is controversial whether Linnean ranks could be conciliated with phylogenetic-based taxonomies (Kuntner & Agnarsson, 2006). For simplicity, in the discussion of the topologies found here, I follow the convention to name informal lineages, defined topologically and anchored to the genus names of the non-Tunisian taxonomic units included in the analysis. Accordingly, for *“Genus name A” lineage* it is meant the most inclusive lineage including the non-Tunisian taxonomic unit(s) referred in literature to Genus *A* and excluding all other taxonomic units referred in literature to other genus-level Linnean ranks. These lineages are meant exclusively as clades and even if mention “genus-level” taxa, they do not refer to particular Linnean ranks. For example, the term “*Asiatoceratodus* lineage” refers to the most inclusive lineage resulted by the analyses performed here that includes the two non-Tunisian specimens HGS 64 and UFMA 1 40 454 (both referred in literature to *Asiatoceratodus*, see Fanti et al., 2016a and references therein) and excludes all other non-Tunisian specimens analyzed.

I performed Bayesian phylogenetic analysis to a modified version of the character-taxon matrix of Fanti et al. (2016a), integrating the morphological data with chronostratigraphic information, following the methods discussed by Lee et al. (2014a), Lee et al. (2014b) and Gavryushkina et al. (2016) (see model settings below). Modifications of the original character-taxon matrix involved:

(1) The removal of one of the two outgroup taxa included in the parsimony analysis of Fanti et al. (2016a), QMF 2108, referred to the Lower Cretaceous ceratodontid *Metaceratodus wollastoni*, and the use of a single taxonomic unit, ZPAL ABbIII 2393, referred to *Ptychoceratodus roemeri*, as outgroup. This operational taxonomic unit is Late Triassic in age and is considered as a more appropriate representative of the ancestral morphology for the ingroup than QMF 2108, from both phylogenetic and stratigraphic reasons, because it consistently pre-dates all other included taxonomic units. The Early Cretaceous age of QMF 2108 implies a >50 Mys long branch for this terminal unit relative to the root of the tree (the latter must be older than the Triassic terminal ZPAL ABbIII 2393): as outlined by Lee et al. (2014a), younger terminal units may have undergone more morphological anagenesis than older units, with the consequence that it cannot be dismissed that the character state combination in QMF 2108 had significantly diverged from the ancestral combination at the root relative to ZPAL ABbIII 2393.

(2) The multistate character statement #3 was split into two binary character statements (i.e., the redefined character #3 and the new character #43; see Appendix S1).

(3) The character statement #8 was defined as binary instead of multistate: the previous state “2” in character #8 in Fanti et al. (2016a) is clearly redundant with state “1” of character #9 (i.e., an angled mesial margin defines two distinct mesio-buccal and mesio-internal margins). Accordingly, the previous states “1” and “2” of character #8 were merged into a single state “1” as both describe the same condition, i.e., a convex mesial margin (see Appendix S1).

(4) *A priori* removal of characters #2, #7 and #10 as they refer to measurement values of tooth plate margins. Exploration of the character scores in the original matrix shows that these three characters co-vary consistently. Thus, these character statements are redundant, referring to the same phenomenon (the absolute size of the plate). Furthermore, size-based characters are individually- and ontogenetically-variable features with poor phylogenetic signal.

Modifications (2) and (3) have removed all the redundant character statements present in the parsimony analysis (Fanti et al., 2016a) and have replaced the non-redundant multistate characters with a series of analogous binary character statements. In particular, this modification results in the included character #3 as being split into two binary characters (the new #3 and the #46). One reason for splitting multistate character statements into a series of simpler binary characters is to allow the Bayesian analysis to test whether different state transitions evolved at different rates. In parsimony analysis, different state transitions along the evolution of a feature occur at the same rate regardless of being all states of the same character or being them split into distinct character statements. On the contrary, in likelihood analyses using the rate variability gamma parameter, different state transitions can evolve at different rates if they are defined as distinct characters. Thus, splitting a multistate character included in a Bayesian inference phylogenetic analysis into a series of non-redundant binary characters allows to investigate the effect of among-state variation heterogeneity in the evolution of that character.

Bayesian analyses were performed using BEAST (Bayesian Evolutionary Analysis Sampling Trees) vers. 2.4.4 (version updated in November 2016, Drummond et al., 2012; Bouckaert et al., 2014). Usually, in phylogenetic analyses based on morphological characters and using parsimony as tree search strategy, only variable characters (potential synapomorphies) are sampled (Lewis, 2001; Lee et al., 2014a). Being all the terminal units used in this analysis represented by single individuals, the term “autapomorphy” for those character states present exclusively in a single terminal unit is probably misleading: features that are autapomorphies at the species-level are recorded as synapomorphies at the specimen-level among conspecific individuals. Thus, “terminal” feature is here preferred over “autapomorphy” when referring to a character state change optimised along a specimen-level tip. The original character statements used in the analysis of Fanti et al. (2016a) were based on a series of phylogenetically significant features, mostly derived from the literature and suggested to diagnose “genus/species-level” taxa, including characters with a high level of homoplasy (in particular, characters that may not result synapomorphic at any node but may result as terminal features in two or more distinct terminal branches). It is here assumed that the terminal features may provide information on the length of the terminal branches in an analogous way as autapomorphies for species-level tips. In the analysis performed here, the Markov-Chain Monte Carlo Bayesian method for estimating phylogeny used the Lewis’s (2001) Markov model for the evolution of discrete morphological characters. Variability in rates of evolution among characters was accomodated using the gamma distribution, and variability across lineages was accomodated using the relaxed clock model (Lee et al., 2014b, supplementary material; Dembo et al., 2015). All characters were treated as a single partition, and the Lewis’s (2001) model was conditioned to variable characters only using the implementation included in BEAST vers. 2.4.4. The Fossilized Birth-Death model with Sampled Ancestors implemented by Gavryushkina et al. (2016) was used as tree prior. In this study, the only notable difference from the method used by Gavryushkina et al. (2016) was the setting of the rho parameter, that defines the probability of sampling at the present: being the analyzed sample formed exclusively by fossil individuals, rho was set as  = 0.

A significant application of Bayesian inference in phylogenetic analysis of fossil taxa compared to parsimony analysis is the integration of morphological and stratigraphic (age) information during tree search (Lee et al., 2014a; Lee et al., 2014b). Absolute age ranges were determined for each terminal unit (based on Fanti et al., 2016a) according to the ages reported in the International Chronostratigraphic Chart (International Commission on Stratigraphy, vers. 2016; http://www.stratigraphy.org). In absence of direct dating from radiometric analysis, the absolute age of fossil taxa is usually inferred from the age of the boundaries of the stratigraphic series including those taxa (Lee et al., 2014a), which implies a variable amount of uncertainty on the age of the tip. In order to incorporate age uncertainty in the analysis, the ages of each terminal tip included in this study were defined as uniform range priors instead of using single (mean) values. In particular, the ages of all Tunisian specimens were conservatively set along an uniform range sampling the whole Albian stage (∼113–100 Mya). The age of the two most recent operational taxonomic units included (ROM 47626 and ROM 47627, both referred to *Lavocatodus humei*, see Fanti et al., 2016a) were both fixed at 83 Mya (the mean value between the lower and upper boundary ages of the Late Cretaceous, see Fanti et al., 2016a, supplementary material), because BEAST vers.2 requires at least the age of the most recent terminals to be fixed.

The BEAST analysis involved five replicate runs (with different random starting trees and random number seeds). Each of the replicate runs involved 50 million steps with sampling every 5,000 generations, with a burn-in set at the first 20% sampled. The Log and Tree output files of the five replicates were merged using LogCombiner (Drummond et al., 2012; Rambaut & Drummond, 2009). Convergence (stationarity) in numerical parameters was identified using Tracer vers. 1.5 (Rambaut & Drummond, 2009). The Maximum Clade Credibility Tree (MCCT) resulted from the Bayesian analysis was used as a temporally-calibrated phyletic framework for phylogenetic and taxonomic discussion.

In order to test whether the clades including the Tunisian specimens are locality-specific, the four Tunisian localities where the specimens have been collected (i.e., El Hmaima, El Kambout, El Mra, Oum ed Diab; see Fanti et al., 2016a, supplementary information) were plotted on the resulted phylogenetic framework. The MCCT resulted from the Bayesian analysis was used as a temporally calibrated phyletic framework for palaeobiogeographic reconstruction, inferring ancestral geographic placement of nodes using RASP (Reconstruct Ancestral State in Phylogenies, Yu et al., 2015). The distribution range of the taxonomic units was *a priori* divided into five areas: “Non-Tunisia” (all non-Tunisian specimens were scored for this area, used as paleogeographic outgroup for the analysis), El Mra, Oum ed Diab, El Kambout, and El Hmaima. Each terminal taxon was scored for the area character state according to the location where it was recovered. Locality inferences on the phylogenetic frameworks were obtained in RASP by applying Bayesian Binary Markov Chain Monte Carlo (BBM) analysis (Yu et al., 2015). The BBM method suggests possible ancestral ranges at each node and also calculates probabilities of each ancestral range at nodes according to both tip scores and branch lengths. The BBM analyses performed ten Markov Chain Monte Carlo chains of 50,000 cycles each, sampling every 100 trees. Chain temperature was set at 0.1. State frequencies were set as estimated and among-site rate variation was set using the gamma parameter. The first 20% of the recovered trees were discarded and the remaining trees were used to infer ancestral range distribution at nodes. The time-events algorithm implemented in RASP (Yu et al., 2015) was used to infer the total distribution of cladogenetic events at the El Mra and Oum ed Diab localities (where the majority of the Tunisian specimens has been collected) along the chronologic interval estimated by the BEAST analysis.

## Results

**Figure 1:**
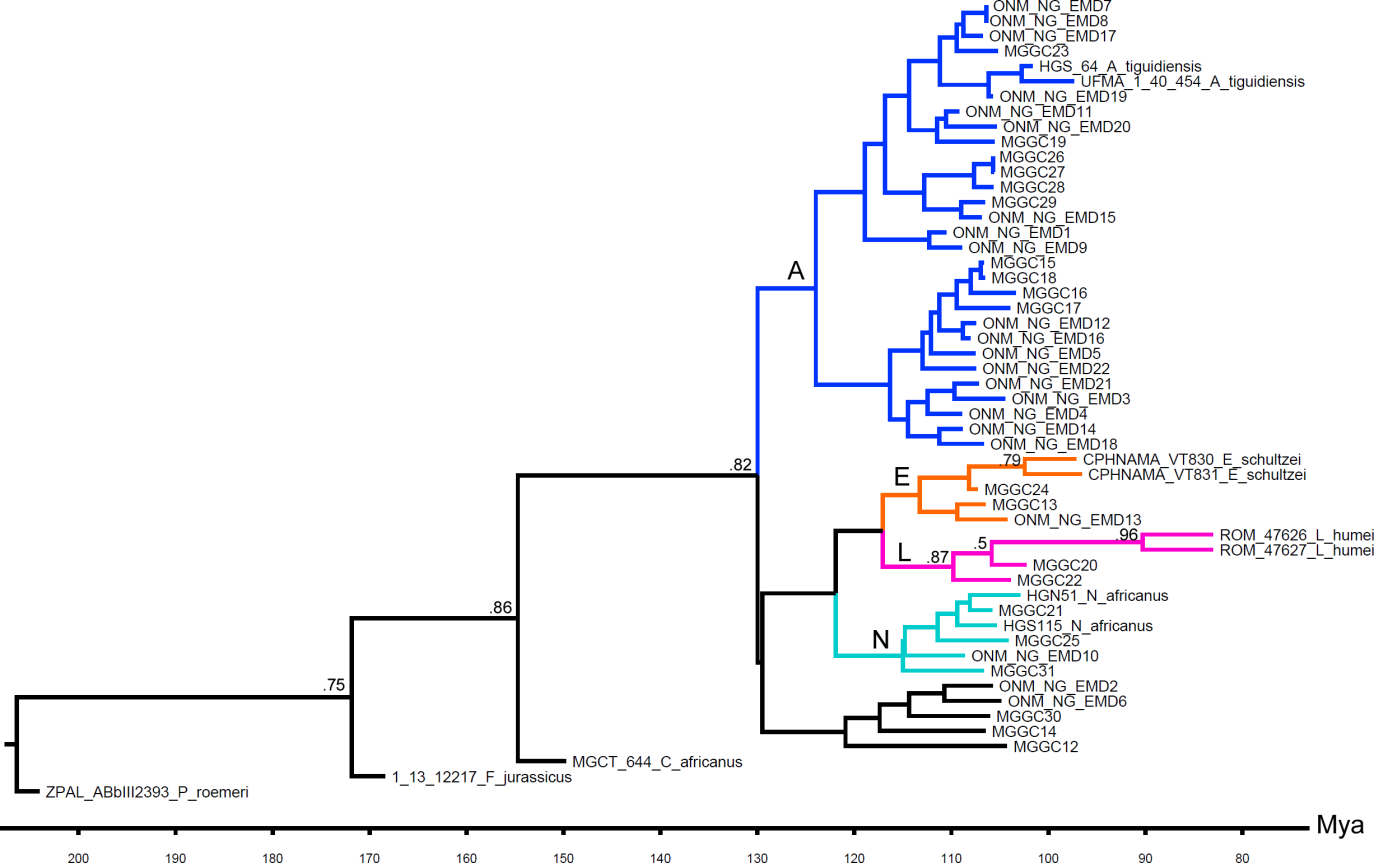
MCCT resulted by Bayesian phylogenetic analysis of the dipnoan specimens discussed in this study. Numbers adjacent to nodes indicate posterior probability value greater than or equal to 0.5. Abbreviations: A, *Asiatoceratodus* lineage; E, *Equinoxiodus* lineage; L, *Lavocatodus* lineage; N, *Neoceratodus* lineage.

**Figure 2:**
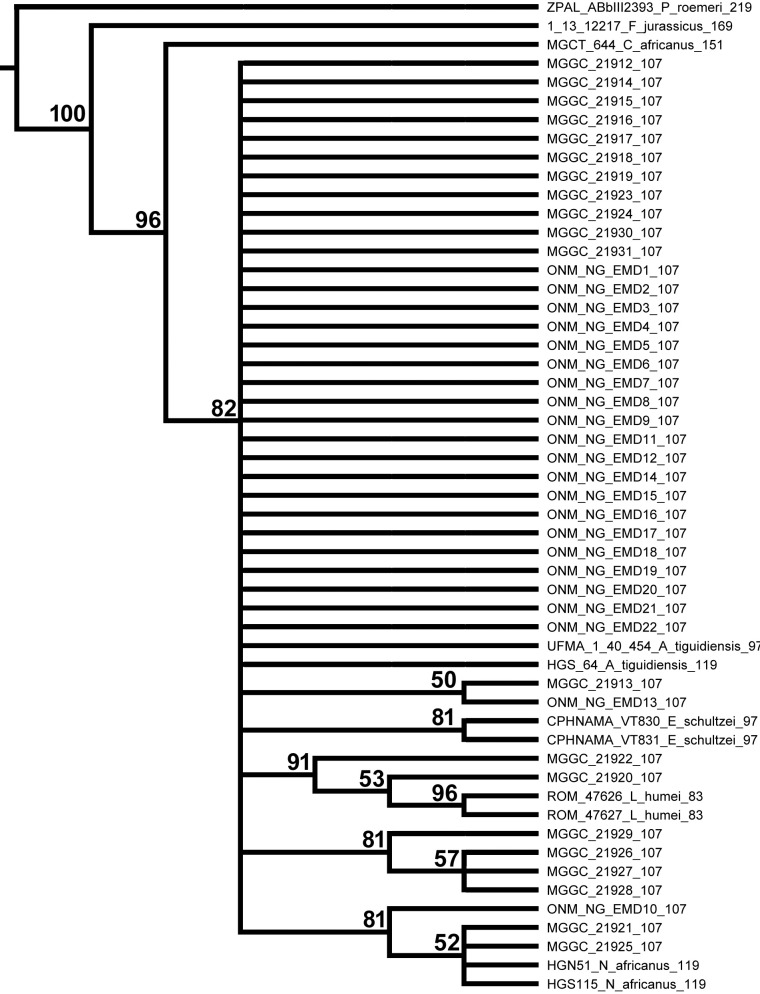
Half compact (majority rule) consensus of the topologies found among the post-burnin trees saved. Branch lengths not to scale. Numbers at end of terminal unit names indicate mean value of tip priors (in Mya).

The MCCT of the combined tree samples supports the monophyly of the non-Tunisian species included in the analysis (Fig. 1): each least inclusive node containing the representatives of these species does not include any member of the other species. Convergence (stationarity) in parameters identified using Tracer vers. 1.5 (Rambaut & Drummond, 2009) is supported by effective sample size (ESS) of every parameter being >200. Focusing on the MCCT topology, the analysis found *Ferganoceratodus jurassicus* as the basalmost lineage of the ingroup (posterior probability, *pp*, value is 0.75), as sister-taxon of the node containing the specimen of *Ceratodus africanus* and a clade including all other specimens (*pp*: 0.86). The latter clade (*pp*: 0.82) is formed by two main lineages: the most inclusive, leading to the specimens of *Asiatoceratodus* cf. *tiguidensis*, and the other including the specimens referred to *Neoceratodus africanus*, *Lavocatodus humei* and *Equinoxiodus schultzei*. The robustness of the higher-level relationships among the main lineages including the Tunisian specimens is very low (*pp* < 0.5) for the majority of nodes, and most of these nodes are recovered in less than half of the sampled trees (Fig. 2). Nevertheless, this is expected because the evaluation of ceratodontid higher-level relationships was beyond the aims of this study, and the data matrix was assembled to test lower-level relationships using exclusively tooth plate features. Among the main lineages recovered in the MCCT, the analysis found support for the referral of specimen MGGC 21920 and MGGC 21922 to the *Lavocatodus* lineage (*pp*: 0.87). A subset of the Tunisian specimens is recovered among a lineage that is sister taxon of the clade including the *Equinoxiodus*, *Lavocatodus* and *Neoceratodus* lineages, but does not lead to non-Tunisian specimens.

Bayesian analysis integrating morphological and stratigraphic information simultaneously estimates relationships among clades and the timing of cladogenesis (Lee et al., 2014a; Lee et al., 2014b). Based on the median age of the nodes in the MCCT, the lineage leading to all ingroup specimens diverged from the lineage leading to *Ptychoceratodus roemeri* in the Late Triassic (mean age: ∼206 Mya). The mean age of divergence of the lineage leading to the specimen referred to *Ferganoceratodus* from its sister lineage is ∼172 Mya, and the divergence of the lineage leading to *Ceratodus africanus* specimen from the lineage including all other specimens is inferred at ∼155 Mya. The mean age of the last common ancestor of all Tunisian specimens included in the analysis is inferred at ∼130 Mya. In the MCCT, all the terminal branches leading to the Tunisian specimens have been inferred to originate between 121 and 106 Mya.

**Figure 3:**
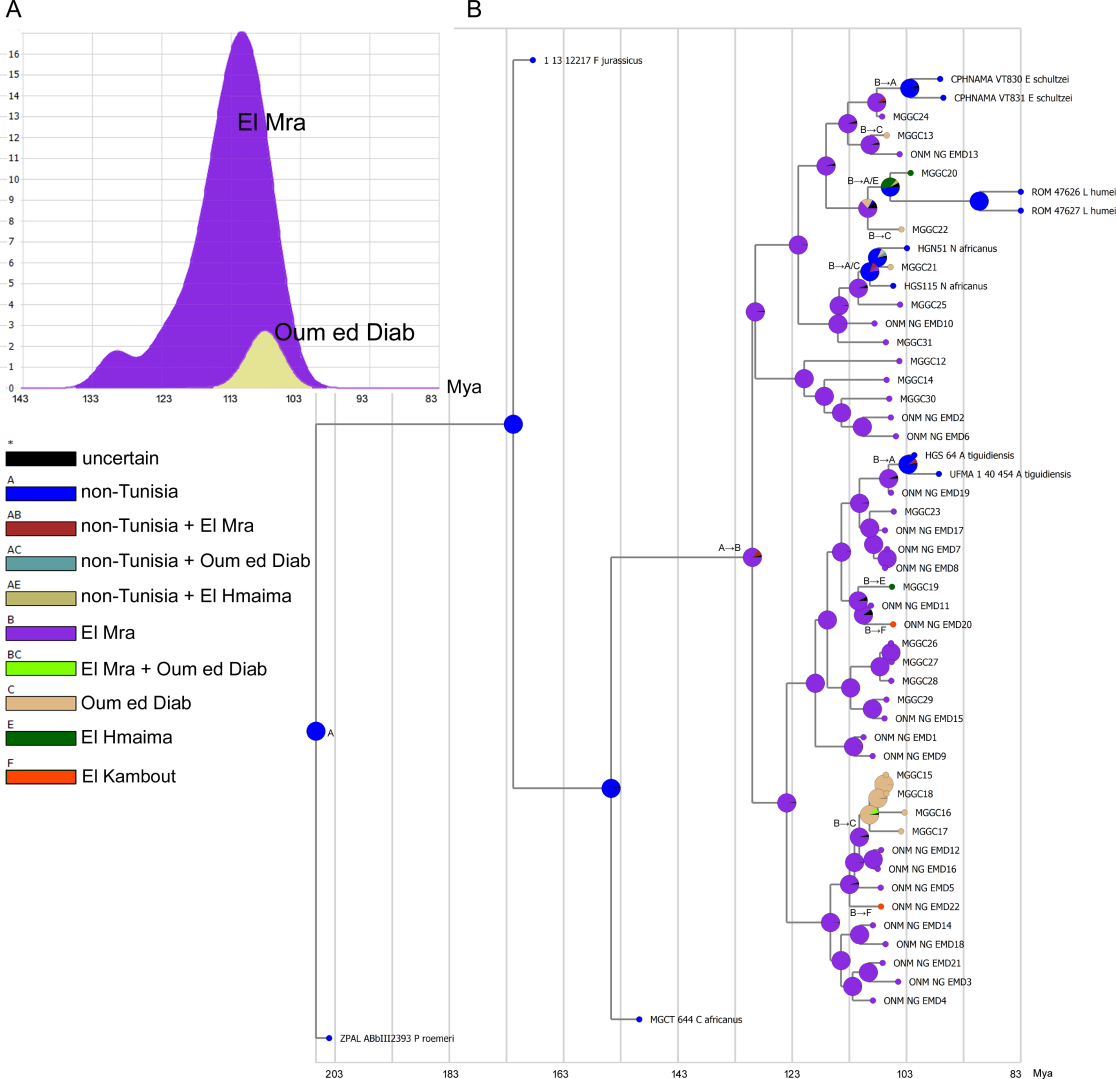
Stratigraphic inference from the MCCT framework. (A) Result of the time-events algorithm analysis using RASP for the El Mra and Oum ed Diab localities, showing the number of cladogenetic events inferred at El Mra and Oum ed Diab. (B) Ancestral Area Reconstruction at the locality-scale using the framework obtained by the phylogenetic analysis using the BBM method in RASP. Migration events indicated at nodes (e.g., “A → B” indicates a migration from area A to area B).

The use of the FBD model in tree reconstruction allows to test whether one or more members of the analyzed ingroup can be ancestor(s) of other sampled taxa and formed anagenetic series. Exploration of the relationships found among the post-burnin trees saved indicates that the median number of sampled ancestors *per* topology sampled is 7 (95% CI [0–14]).

When the sampled localities are plotted on the MCCT diagram (Fig. 3), all the four main lineages including the Tunisian specimens are represented at the El Mra locality. The two specimens from El Hmaima (MGGC 21919 and 21920) resulted, respectively, one among the *Asiatoceratodus* lineage as sister taxon of one of the two specimens from El Kambout, and the other as sister taxon of the lineage leading to the two non-Tunisian specimens of *Lavocatodus humei*. The other specimen colleted at El Kambout resulted a member of the *Asiatoceratodus* lineage. Among the specimens collected at the Oum ed Diab locality, four formed a clade that is nested among the *Asiatoceratodus* lineage. The other three specimens from Oum ed Diab resulted, respectively, each among the *Equinoxiodus*, *Lavocatodus* and *Neoceratodus* lineages. All the other specimens were collected from El Mra and are referable to the four main lineages. The BBM analysis of the locality distributions relative to the phyletic framework inferred El Mra as the ancestral area for the last common ancestor of the Tunisian sample and for most of the lineages of the sample, and Oum ed Diab as the ancestral area for a subclade of the *Asiatoceratodus* lineage (the sample from the other localities is too small to be analyzed). The time-events algorithm implemented in RASP was used to estimate the number of cladogenetic events inferred to be recorded at the two localities according to the phylogenetic framework. This test is used to compare the richness of the fossil record from El Mra relative to that from Oum ed Diab, assuming that, given the relative geographic proximity and lateral continuity between the two series (Fanti et al., 2016a) the difference in their taxonomic disparity is mostly due to depositional and taphonomic factors than a genuine evolutionary signal, and the more inclusive the stratigraphic series is the larger number of cladogenetic events are documented there. The time-events algorithm test for the two localities suggests that the currently known fossil record from Oum ed Diab is stratigraphically less inclusive than the record from El Mra and overlaps only the youngest part of the record from the latter locality (Fig. 3A). The BBM analysis also revealed a shared pattern among the Tunisian specimens relative to the localities where they were sampled: all the specimens from El Hmaima, El Kambout and Oum ed Diab are nested among clades formed by the specimens from El Mra. Although this result may be partly a sampling artefact, biased by the richer sample from El Mra relative to the other localities, it is noteworthy that the inferred relationships among the localities, according to the MCCT topology, is described by a relatively simple scenario that requires seven migration events, all starting from El Mra (Fig. 3B): three migration events from El Mra to Oum ed Diab, two events from El Mra to El Kambout, two events from El Mra to El Hmaima. None of the specimens from El Mra is interpreted as being the result of migration events started from the other localities.

## Discussion

Fanti et al. (2016a) identified the majority of the specimens included in this sample at the genus- or species-level based on the shared presence of diagnostic features reported in the literature. About 60% of the taxonomic identifications provided by Fanti et al. (2016a) are confirmed by the result of the Bayesian analysis (Table 1). In particular, the identification of all but one specimen of *Asiatoceratodus*, and of all specimens of *Equinoxiodus* and *Lavocatodus* suggested by Fanti et al. (2016a) is confirmed by the result of the Bayesian analysis. All the specimens identified as belonging to *Ceratodus* or *Ferganoceratodus* by Fanti et al. (2016a) have been re-interpreted as belonging to the three above mentioned taxa or to a yet-unnamed lineage. These results suggest that the combinations of tooth plate features used in literature to diagnose the taxa *Asiatoceratodus*, *Equinoxiodus* and *Lavocatodus* are phylogenetically significant and allow an accurate identification of these taxa even using isolated dental elements. On the contrary, the results of the Bayesian analysis do not support the identification of the isolated tooth plates to *Ceratodus* and *Ferganoceratodus*: this suggests that the two taxa cannot be identified from isolated tooth plates, or, alternatively, that the features used in literature to diagnose them define non-monophyletic assemblages. The second interpretation is indirectly supported by the topology of the MCCT (Fig. 1): *Ferganoceratodus* and *Ceratodus* form a paraphyletic series along the basal branch leading to the clade containing the other genus-level taxa and the Tunisian specimens.

**Table 1:** Alternative identifications of the specimens studied.

Specimen	Identification based on apomorphies (Fanti et al., 2016a)	Referral based on parsimony analysis (Fanti et al., 2016a)	Referral based on Bayesian analysis (this study)	Locality
ONM NG EMD 1	*Asiatoceratodus*	Uncertain	*Asiatoceratodus*	El Mra
ONM NG EMD 2	*Ferganoceratodus*	Uncertain	New lineage	El Mra
ONM NG EMD 3	*Asiatoceratodus*	New lineage	*Asiatoceratodus*	El Mra
ONM NG EMD 4	*Asiatoceratodus*	Uncertain	*Asiatoceratodus*	El Mra
ONM NG EMD 5	*Asiatoceratodus*	Uncertain	*Asiatoceratodus*	El Mra
ONM NG EMD 6	*Asiatoceratodus*	Uncertain	New lineage	El Mra
ONM NG EMD 7	*Asiatoceratodus*	Uncertain	*Asiatoceratodus*	El Mra
ONM NG EMD 8	*Asiatoceratodus*	Uncertain	*Asiatoceratodus*	El Mra
ONM NG EMD 9	*Asiatoceratodus*	Uncertain	*Asiatoceratodus*	El Mra
ONM NG EMD 10	*Ferganoceratodus*	*Neoceratodus*	*Neoceratodus*	El Mra
ONM NG EMD 11	*Asiatoceratodus*	Uncertain	*Asiatoceratodus*	El Mra
ONM NG EMD 12	*Asiatoceratodus*	Uncertain	*Asiatoceratodus*	El Mra
ONM NG EMD 13	*Equinoxiodus*	New lineage	*Equinoxiodus*	El Mra
ONM NG EMD 14	*Ceratodus*	Uncertain	*Asiatoceratodus*	El Mra
ONM NG EMD 15	*Ferganoceratodus*	Uncertain	*Asiatoceratodus*	El Mra
ONM NG EMD 16	*Asiatoceratodus*	Uncertain	*Asiatoceratodus*	El Mra
ONM NG EMD 17	*Asiatoceratodus*	Uncertain	*Asiatoceratodus*	El Mra
ONM NG EMD 18	*Ceratodus*	Uncertain	*Asiatoceratodus*	El Mra
ONM NG EMD 19	*Asiatoceratodus*	Uncertain	*Asiatoceratodus*	El Mra
ONM NG EMD 20	*Asiatoceratodus*	Uncertain	*Asiatoceratodus*	El Kambout
ONM NG EMD 21	Uncertain	New lineage	*Asiatoceratodus*	El Mra
ONM NG EMD 22	*Asiatoceratodus*	Uncertain	*Asiatoceratodus*	El Kambout
MGGC 21912	*Ceratodus*	Uncertain	New lineage	El Mra
MGGC 21913	*Equinoxiodus*	New lineage	*Equinoxiodus*	Oum ed Diab
MGGC 21914	*Ceratodus*	Uncertain	New lineage	El Mra
MGGC 21915	*Asiatoceratodus*	Uncertain	*Asiatoceratodus*	Oum ed Diab
MGGC 21916	*Asiatoceratodus*	Uncertain	*Asiatoceratodus*	Oum ed Diab
MGGC 21917	*Asiatoceratodus*	Uncertain	*Asiatoceratodus*	Oum ed Diab
MGGC 21918	*Asiatoceratodus*	Uncertain	*Asiatoceratodus*	Oum ed Diab
MGGC 21919	*Asiatoceratodus*	Uncertain	*Asiatoceratodus*	El Hmaima
MGGC 21920	*Lavocatodus*	*Lavocatodus*	*Lavocatodus*	El Hmaima
MGGC 21921	*Neoceratodus*	*Neoceratodus*	*Neoceratodus*	Oum ed Diab
MGGC 21922	*Lavocatodus*	*Lavocatodus*	*Lavocatodus*	Oum ed Diab
MGGC 21923	*Asiatoceratodus*	Uncertain	*Asiatoceratodus*	El Mra
MGGC 21924	*Ceratodus*	Uncertain	*Equinoxiodus*	El Mra
MGGC 21925	*Neoceratodus*	*Neoceratodus*	*Neoceratodus*	El Mra
MGGC 21926	*Ferganoceratodus*	Uncertain	*Asiatoceratodus*	El Mra
MGGC 21927	*Ferganoceratodus*	Uncertain	*Asiatoceratodus*	El Mra
MGGC 21928	*Ferganoceratodus*	Uncertain	*Asiatoceratodus*	El Mra
MGGC 21929	*Ferganoceratodus*	Uncertain	*Asiatoceratodus*	El Mra
MGGC 21930	*Ferganoceratodus*	Uncertain	New lineage	El Mra
MGGC 21931	Uncertain	Uncertain	*Neoceratodus*	El Mra

The majority of the nodes recovered by the Bayesian analysis of the modified data set of Fanti et al. (2016a) using the FBD model show low posterior probability values (*pp* <0.5). This result is not unexpected, and is due to the low number of phylogenetically significant features obtained from the tooth plate morphology relative to the number of included specimens (43 characters vs. 53 taxonomic units) and the high level of homoplasy among the specimens (Fanti et al., 2016a). Nevertheless, the Bayesian analysis performed here integrated stratigraphic information, not included in the previous analysis using parsimony as tree search strategy (Fanti et al., 2016a), and obtained some relationships with a relatively robust support. In particular, the analysis indicates that the last common ancestor of the sampled specimens from Tunisia was Early Cretaceous in age (∼130 Mya). This topology constraints the origin and evolution of the dipnoan taxa sampled in the Ain el Guettar Formation to a 20–30 Myrs long interval. This result markedly differs from that discussed by Fanti et al. (2016a: Fig. 10) based on parsimony analysis, that estimated at least four lineages leading to the Tunisian specimens that had to be extended back to the Middle Jurassic in order to re-conciliate the phyletic pattern with the stratigraphic placement of some of the non-Tunisian specimens included in the analysis.

In summary, the Bayesian analysis of the dipnoan specimens from the Ain el Guettar Formation does not support the faunal diversity reported by Fanti et al. (2016a). As stated above, *Ferganoceratous* is found to be outside the least inclusive clade containing all Tunisian specimens. Furthermore, none of the specimens sampled has been referred to *Ceratodus* (*contra* the results in Fanti et al., 2016a): the specimens referred by Fanti et al. (2016a) to that genus have been placed by the Bayesian analysis among the basalmost branch of the *Asiatoceratodus* lineage (Table 1). The Bayesian analysis confirms that *Asiatoceratodus* is the most abundant clade, being it found in all localities (Fanti et al., 2016a). The *Equinoxiodus* lineage is found in two localities (El Mra and Oum ed Diab). The *Lavocatodus* lineage is also recorded in two localities, respectively, at El Hmaima (where *Equinoxiodus* is not found) and Oum ed Diab. The *Neoceratodus* lineage is found at El Mra and Oum ed Diab. The 95% confidence ranges of the ages of the terminal tips from the four Tunisian localities inferred by the Bayesian analysis broadly overlap, a result that confirms the lateral equivalence among the series from the four localities (Fanti et al., 2016a). The RASP analysis was used to compare the richness of the fossil record from the El Mra locality relative to that from the Oum ed Diab locality, following the hypothesis that the sections exposed at the two localities were laterally equivalent (Fanti et al., 2016a). Focusing on the MCCT framework and the distribution of the specimens in the localities, the scenario resulted by the RASP analysis suggests that the dipnoan lineages sampled from the El Kambout, El Hmaima and Oum ed Diab localities descended from migration events originated from the El Mra locality. Apparently, the relationships among the sampled localities (i.e., El Mra resulting the ancestral locality for the Tunisian sample inferred by the RASP analysis) and the asymmetrical relationships in the polarity of the migration events among the four localities inferred by the RASP analysis of the MCCT topology (i.e., all migration events started from El Mra) challenge the hypothesis that these localities were laterally equivalent, and may indicate diachrony among these sections. The migration episodes inferred by the RASP analysis could be considered as spurious events, analytical artifacts due to poor sampling from the sections at the El Hmaima, El Kambout, and Oum ed Diab localities. Although artifacts in specimen collection and the non-homogeneous sampling among the localities may explain this pattern (in particular, about 74% of the whole sample was collected at the El Mra locality), the difference in the time-algorithm profiles of the two most sampled localities (Fig. 3A) may also be explained assuming that the stratigraphic sequence sampled at the Oum ed Diab locality is equivalent to only the upper part of the series that is more extensively recorded at El Mra. This alternative interpretation is confirmed by stratigraphic analyses at regional scale: although the El Mra and Oum ed Diab beds represent partially lateral equivalent deposits, the latter locality is representative only of the youngest history of the section (Fanti et al., 2016a and references therein). The lower beds of this unit (mostly recorded at El Mra) are interpreted as fluvial sand bars that deposited in a vast estuarine system, whereas the overlying deposits (recorded at Oum ed Diab) gradually shift to shoreface, tidal flat, and foreshore deposits.

In this study, the FBD model with sampled ancestors (Gavryushkina et al., 2016) has been applied for the first time to a set of exclusively extinct taxa sampled at the specimen level (thus, avoiding *a priori* assumptions on species-level definitions, diagnosis and inclusiveness). One advantage of the FBD model relative to previously developed models for phylogenetic inference is that it allows to test ancestor-descendant relationships among a sample of fossils. The failed recognition of ancestors among a sample of taxonomic units may lead to the inference of spurious cladogenetic events, and to overestimation of the number of co-existing lineages along a particular time interval. Furthermore, over-estimation of cladogenetic events significantly bias the parameter estimation at branches, in particular the estimation of lineage extent and duration (Gavryushkina et al., 2014; Gavryushkina et al., 2016).

The Triassic or Jurassic origins for some of the Tunisian lineages that were inferred by the stratigraphic calibration of the topology resulted by the parsimony analysis in Fanti et al. (2016a), compared to the exclusively Cretaceous ages recovered by the Bayesian analysis using the FBD model here, is probably biased by methodological artifacts, in particular, the use of tree search strategies, like parsimony, that are unable to incorporate stratigraphic information in tree reconstruction. Although the use of the FBD model with sampled ancestors represents a more realistic reconstruction of the evolutionary history of the Ain el Guettar Formation dipnoan specimens compared to the strictly cladogenetic pattern resulted by parsimony analysis (which does not incorporate stratigraphic information during the tree search), it should be remarked that the FBD model assumes uniform rate of sampling for the fossil specimens over time. Nevertheless, the sample analyzed here does not adequately met such assumption, because it is not uniformly distributed over time (i.e., although the whole sample spans from the Late Triassic to the Late Cretaceous, the large majority of the specimens is distributed exclusively in the Albian-Cenomanian stage). Future implementations of the FBD model with sampled ancestors may incorporate heterogeneity in the rate of fossil sampling over time (see Stadler et al., 2013, for an epidemiological application of this approach).

The use of specimens as terminal units instead of species means that the topological pattern recovered in the MCCT may include both intraspecific and interspecific relationships. In particular, intraspecific relationships may indicate genealogic sequences among populations of the same species, or anagenetic sequences along a phyletic lineage without splitting events (Gould, 2002). In this study, the character list was based on morphological features previously used for species/genus-level identifications among Mesozoic ceratodontids, and it is not unexpected that the most robust relationships found by the Bayesian analysis are among the nodes that support supraspecific relationships, whereas the intraspecific relationships result relatively weakly supported. The incorporation of age uncertainty (tip priors) in the FBD model allows the analysis to simulate anagenetic series among the specimens from the same stratigraphic series because tip-dates were treated as random variables with uniform prior distributions, with bounds based on the shortest chronostratigraphic range including the Ain el Guettar Formation. These anagenetic series are retained in the saved trees if they fit the data (in particular, the morphological information) better than a strictly cladogenetic pattern.

## Conclusions

Phylogenetic analysis integrating morphological and statigraphic information and using the Fossilized Birth-Death model implemented by Gavryushkina et al. (2016) was applied to investigate the diversity among a sample of isolated specimens referred to dipnoan sarcopterygians from the Ain el Guettar Formation. The analysis estimated an earliest Cretaceous age for the last common ancestor of the Tunisian sample and provided a framework for comparing the taxonomic composition of the samples from distinct localities at the Ain el Guettar Formation. Previous analyses using parsimony suggested five or more genus-level lineages included in this Tunisian sample (Fanti et al., 2016a). In particular, Fanti et al. (2016a) included *Ceratodus* and eventually *Ferganoceratodus* among the lineages represented in the sample, a result not supported by the Bayesian analysis performed here. The taxonomic content of the four Tunisian localities sampled is not homogeneous. Although sampling artifacts cannot be dismissed among the factors producing this taxonomic heterogeneity, comparison between the phylogenetic pattern resulted and the geographic distribution of the specimens among the sampled localities supports the hypothesis that the El Mra locality represents a stratigraphic sequence more inclusive than the other localities. This interpretation is in agreement with the stratigraphic analysis of the sampled localities along the Oum ed Diab Member, which indicates that the largest part of the series is recorded at El Mra (Fanti et al., 2016a). In the previous analysis of the sample, Fanti et al. (2016a) suggested that the high taxonomic diversity among the Ain el Guettar dipnoans was inflated by taphonomic artifacts. Although this study does not dismiss some role for taphonomic factors in inflating the diversity recovered in the Ain el Guettar Formation (Fanti et al., 2016a), it is suggested that the taxonomic diversity of fossil assemblages may be inflated by analytical approaches not taking into account the stratigraphic information or the presence of anagenetic lineages (see Fanti et al., 2014).

**Figure 4:**
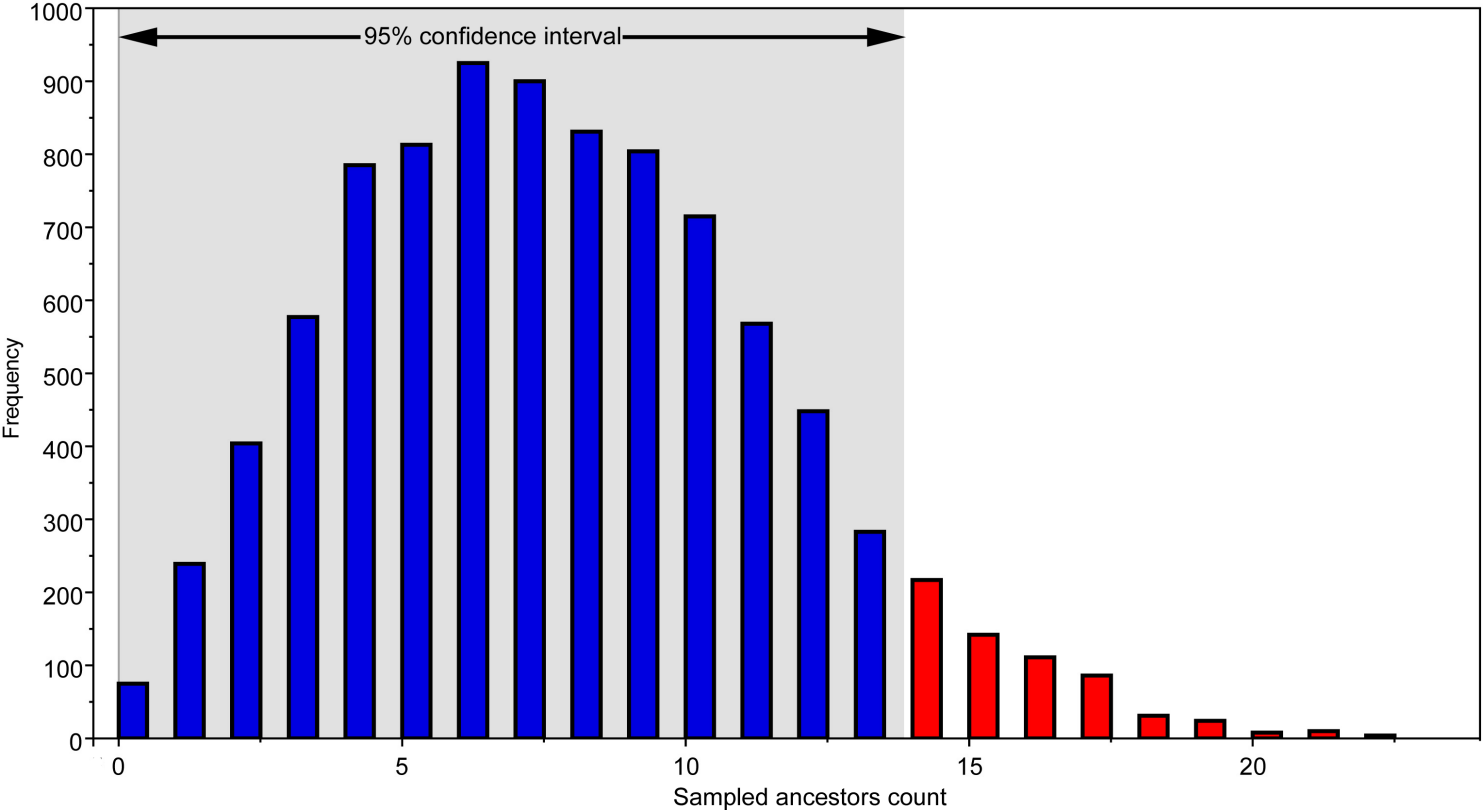
Frequency of sampled ancestors counted in the post-burnin trees recovered. Arrow indicates the 95% confidence interval.

The FBD model with sampled ancestors and incorporating tip priors for the analysis of fossil taxa may constitute a novel approach not only because it integrates morphological and stratigraphic information in macroevolutionary and systematic analysis of higher-level clades, but also as a methodology for lower-level taxonomic analysis using specimens and individuals as terminal units instead of species. As a method discriminating anagenetic lineages from cladogenetic patterns, the FBD model, and in particular the approach used here incorporating tip age uncertainty, may improve our knowledge of those phenomena at the boundary between micro- and macroevolution (Gould, 2002). The recognition of ancestor-descendant relationships in fossils is debated (Szalay, 1977; Bretsky, 1979; Dayrat, 2005; Scannella et al., 2014). In this study, 95% of the sampled trees include a number of sampled ancestors ranging between 0 and 14 (median value, 7; Fig. 4). This value suggests that up to 23% of the specimens collected in the sample may represent members of populations that are anagenetic ancestors of the other individuals included. As noted above, failed recognition of potential ancestors may led to overestimate the number of lineages represented in a fossil assemblage. The application of the FBD model with sampled ancestors and incorporating tip age uncertainty to a broad series of fossil clades may help in estimating the frequency of ancestor-descendant relationships in the fossil record. Furthermore, this method may also represent an auxiliary tool for testing hypotheses on the taxonomic diversity among stratigraphically related localities.

##  Supplemental Information

10.7717/peerj.3055/supp-1 Supplemental Information 1 Data file for analysis in BEAST vers.2

10.7717/peerj.3055/supp-2 Appendix S1 Character statements used in phylogenetic analysis

## Abbreviations

CPHNAMA: Centro de Pesquisa de História Natural e Arqueologia do Maranhão, Praia Grande, São Luís, Brazil
HGN: Museum National d’Histoire Naturelle, Paris, Nord du Hoggar
MGGC: Museo Geologico Giovanni Capellini, Bologna, Italy
MGCT: Museo de Geociencias, Tacuarembó, Uruguay
ONM: Office National des Mines, Tunis
QM: Queensland Museum, Brisbane, Australia
ROM: Royal Ontario Museum, Toronto, Canada
UFMA: Coleção Paleontológica da Universidade Federal do Maranhão, Bacanga, São Luís, Brazil
ZPAL: Institute of Paleobiology, Polish Academy of Sciences,Warsaw, Poland.

## Additional Information and Declarations

### Data Availability

The following information was supplied regarding data availability: The raw data has been supplied as a Supplementary File.
