# Supplementary material for: Specimen-level phylogenetics in paleontology using the Fossilized Birth-Death model with sampled ancestors
Source: PeerJ. 2017 Mar 1;5:e3055. doi: 10.7717/peerj.3055 (PMC5335686; doi:10.7717/peerj.3055)
Supplement: Appendix S1 [file peerj-05-3055-s002.doc]

**APPENDIX**

**Character statements of the morphological features used in the phylogenetic analysis. Number adjacent to character description refers to numeration in Fanti *et al.* (2016a).**

| 1 | 1. Tooth plate morphology, shape (based on lingual margin): triangular, lingual margin uniformly converges to mesointernal angle (0); trapezoidal, lingual margin angled, forming a wide platform labial to the mesointernal angle (1). |
| --- | --- |
| 2 | 3. Cusps, number: 4 (0); more than 4 (1). |
| 3 | 4. Buccal crest, morphology: acute (0); not acute, rounded (1). |
| 4 | 5. Buccal sulci, morphology: “V”-shaped (0); “U”-shaped (1). |
| 5 | 6. Ridges, inner margin length compared to outer margin: subequal or longer (0); shorter (1). |
| 6 | 8. Mesial margin, profile shape: straight (0); convex (1). |
| 7 | 9. Mesial angle, distinction between mesio-buccal and mesio-internal margins: absent (0); present (1). |
| 8 | 11. Lingual margin, shape: straight to concave (0); convex (1). |
| 9 | 12. Inner (mesio-internal) angle: less (0); more (1) than 115°. |
| 10 | 13. Inner (mesio-internal) angle, placement: anterior (0); median (1). |
| 11 | 14. Occlusal ridges, development: thick (0); thin (1). |
| 12 | 15. Occlusal ridges origin: do not originate (0); originate (1) at the inner angle as extensions of the labial margin. |
| 13 | 16. Second occlusal ridge, terminal placement: not at (0); at (1) the interdental margin. |
| 14 | 17. Occlusal ridges paralleling each other: absent (0); present (1). |
| 15 | 18. Lateral keels: absent (0); present (1). |
| 16 | 19. First occlusal ridge, inclination: not aborally (0); aborally (1). |
| 17 | 20. Occlusal ridges, apex, shape: not inclined (0); inclined posteriorly (1). |
| 18 | 21. Occlusal ridges, orientation relative to labial margin: not perpendicular (0); sub-perpendicular (1). |
| 19 | 22. Occlusal furrows, shape: “V”-shaped (0); “U”-shaped (1). |
| 20 | 23. Occlusal furrows, width: wide (0); narrow (1). |
| 21 | 24. Occlusal furrows, shape of inner margin: not rounded (0); rounded (1). |
| 22 | 25. Occlusal furrows, depth: shallow (0); deep (1). |
| 23 | 26. First occlusal furrow, size relative to following sulci: comparable (0); wider, less acute (1). |
| 24 | 27. First couple of occlusal furrow, depth compared to the following furrow: not different (0); deeper and narrower (1). |
| 25 | 28. First occlusal furrow is much incised than the second: absent (0); present (1). |
| 26 | 29. Occlusal furrows, length: not as long as wide (0); as long as wide (1). |
| 27 | 30. Occlusal surface, buccal view, concavity: absent (0); present (1). |
| 28 | 31. Occlusal surface, dotted texture: absent (0); present (1). |
| 29 | 32. Occlusal surface, rounded concavities: absent (0); present (1). |
| 30 | 33. Petrodentine: absent (0); present (1). |
| 31 | 34. Petrodentine, morphology: webbed (0); anostomosing (1). |
| 32 | 35. Furrows, shallow fossae: absent (0); present (1). |
| 33 | 36. Shallow elongate fossae on the lingual margin and more oval on the furrows: absent (0); present (1). |
| 34 | 37. Elongate fossae oriented perpendicularly to the labial margin: absent (0); present (1). |
| 35 | 38. Small rounded pits on the furrows: absent (0); present (1). |
| 36 | 39. Depressions on the furrows bases and on the labial margin articulation: absent (0); present (1) |
| 37 | 40. Random pattern of pits and depressions on the occlusal furrows: absent (0); present (1). |
| 38 | 41. Cusps in lateral view, shape: keel (0); knee-shaped (1). |
| 39 | 42. Upper dentition, pterygo-palatine processes fused to the lingual margin: absent (0); present (1). |
| 40 | 43. Lower dentition, pre-articular: absent (0); present (1). |
| 41 | 44. Pre-articular, number of fossae: single “V”-shaped (0); two (1). |
| 42 | 45. Symphyseal articulations joined to the labial margin: absent (0); present (1). |
| 43 | New. Number of cusps: five or less (0); six or more (1). Note: this character is equivalent to a second state in character 2, set as ordered. The states have been split to analyze different rates in the transition along the different states. |
